# Did changing primary care delivery models change performance? A population based study using health administrative data

**DOI:** 10.1186/1471-2296-12-44

**Published:** 2011-06-03

**Authors:** R Liisa Jaakkimainen, Jan Barnsley, Julie Klein-Geltink, Alexander Kopp, Richard H Glazier

**Affiliations:** 1 Institute for Clinical Evaluative Sciences, Toronto, Ontario, Canada; 2 Department of Family and Community Medicine, University of Toronto, Ontario, Canada; 3 Department of Health Policy, Management and Evaluation, University of Toronto, Ontario, Canada

## Abstract

**Background:**

Primary care reform in Ontario, Canada started with the introduction of new enrollment models, the two largest of which are Family Health Networks (FHNs), a capitation-based model, and Family Health Groups (FHGs), a blended fee-for-service model. The purpose of this study was to evaluate differences in performance between FHNs and FHGs and to compare performance before and after physicians joined these new primary care groups.

**Methods:**

This study used Ontario administrative claims data to compare performance measures in FHGs and FHNs. The study population included physicians who belonged to a FHN or FHG for at least two years. Patients were included in the analyses if they enrolled with a physician in the two years after the physician joined a FHN or FHG, and also if they saw the physician in a two year period prior to the physician joining a FHN or FHG. Performance was derived from the administrative data, and included measures of preventive screening for cancer (breast, cervical, colorectal) and chronic disease management (diabetes, heart failure, asthma).

**Results:**

Performance measures did not vary consistently between models. In some cases, performance approached current benchmarks (Pap smears, mammograms). In other cases it was improving in relation to previous measures (colorectal cancer screening). There were no changes in screening for cervical cancer or breast cancer after joining either a FHN or FHG. Colorectal cancer screening increased in both FHNs and FHGs. After enrolling in either a FHG or a FHN, prescribing performance measures for diabetes care improved. However, annual eye examinations decreased for younger people with diabetes after joining a FHG or FHN. There were no changes in performance measures for heart failure management or asthma care after enrolling in either a FHG or FHN.

**Conclusions:**

Some improvements in preventive screening and diabetes management which were seen amongst people after they enrolled may be attributed to incentive payments offered to physicians within FHGs and FHNs. However, these primary care delivery models need to be compared with other delivery models and fee for service practices in order to describe more specifically what aspects of model delivery and incentives affect care.

## Background

It has been increasingly recognized that health care systems with a strong primary care component are more efficient and better able to handle current and future health care pressures [1-3]. This has led to several primary care reform strategies in the United Kingdom (UK), Australia, the United States (US) and Canada. Common to all of these reform strategies is a movement away from providing service based on a fee-for-service payment system to a more blended payment mechanism which includes incentives for improving quality and performance.

In the late 1990s, the National Health Services (NHS) in the UK refocused health care delivery through the development of Primary Care Trusts which became responsible for budgets of all hospital, community and general medical services [4]. At the same time there was the formation of the National Institute for Health and Clinical Excellence which established clinical guidelines on the appropriate care for people with specific disease conditions. In 2004, the NHS introduced pay-for-performance contracts for family physicians (FPs). On this system, a graduated scale of payments is provided in proportion to an achieved benchmark of a quality of care indicator. Additional NHS reforms in 2010 empower FPs with health care spending and change the emphasis of performance measures to clinical outcomes.

Similarly in Australia, the Practice Incentives Program (PIP), started in 1998, was an effort to support quality improvement activities [5]. Over the years this program has evolved to include a range of outcome-based performance incentives and disease specific incentives.

Health care reform drives many debates under the current political administration in the US. Included in this debate are various physician incentives which pay for quality rather than quantity in healthcare. The reform document signed in March 2010 encourages the implementation of physician payments which enhance primary care services to improve quality of care, mostly within the Medicare population [6].

Canada started to reform its primary care delivery system after the release of the Romanow report in 2002 [7]. In Ontario, the largest province in Canada, the Ministry of Health and Long-Term Care (MOHLTC) introduced two new primary care enrollment models. Family Health Groups (FHGs) are a blended fee-for-service model. The FHGs offer enhanced fee-for-service payments and new billing codes. This includes service enhancement fees for having patients who meet benchmark targets for cervical, breast and colorectal cancer screening. In 2006, FHGs were eligible to receive an annual diabetes (DM) management incentive payment of $60. For this fee FPs within a FHG need to complete a MOHTLC DM flowsheet which documents several required elements (medications, ophthalmology screening, laboratory testing) for their DM patients. This DM flowsheet includes elements of DM management consistent with the Canadian Diabetes Association 2003 Clinical Practice Guidelines. FHNs are a blended capitation-based model. The FHNs include a base payment per patient for the provision of comprehensive care (capitation) plus incentives, premiums and bonuses for preventive care and some chronic disease management. While the FHN model includes the same service enhancement fees for cervical, breast and colorectal cancer screening as FHGs, there are additional reminder fee payments within the FHN model to contact patients for cervical, breast and colorectal cancer screening. The FHN models received the same annual DM incentive payment for the completion of a flowsheet. In 2008, an annual heart failure (HF) management incentive was introduced for both FHGs and FHNs, similar to the DM incentive. There are no incentive payments for the management of any other chronic diseases.

While information is emerging on the impact of different payment mechanisms on physician behavior [8], primary care reform in Ontario, Canada has provided a natural experiment to assess the impact of a capitation-based remuneration system (FHN) to a fee-for-service system (FHG). It also prompts an examination within both FHGs and FHNs of the introduction of incentives (for preventive care and some chronic disease management).

In Canada, performance measurements and quality of care indicators have been developed for the attributes and components of primary care medicine [9,10]. The application of these measures are wide ranging and serve to provide valuable feedback on improving quality care, identifying care deficits in vulnerable populations, and provide information to policy makers on program planning. Existing health administrative data have been a source of information for several preventive care and chronic disease performance measurements in primary care. In fact, several provinces have already published this information [11,12]. Already in Canada, administrative based performance measures for diabetes care have been feedback to family physicians as part of the initiatives to improve chronic disease management [13].

This study compared a capitation-based remuneration model to a fee-for service-based model using Canadian health administrative data to measure key primary care screening and chronic disease performance indicators. The preventive care measures include cervical cancer, breast cancer and colorectal cancer screening. The chronic diseases include heart failure (HF), diabetes (DM) and asthma. The specific objectives were: 1) to provide a cross-sectional comparison of physician performance in FHNs and FHGs; and 2) to compare performance before and after physicians joined these first new primary care groups.

## Methods

### Study Design

This is a cross-sectional study of performance measures amongst FHG and FHN physician practices. This is also a before-after study of performance measures for FHG and FHN physicians.

### Data Sources

Physician demographic data came from the Corporate Provider Database (CPDB) which provides this information on all practicing physicians in Ontario. Patients rostered to a physician participating in a FHG or FHN were identified using the Client Agency Program Enrolment (CAPE) tables. Information on patient age, sex and place of residence was obtained from the Ontario's Registered Persons Database (RPDB) which is the province's health care registry. All residents of Ontario, Canada receive coverage from the Ontario Health Insurance Program (OHIP). Office, home and long-term care visits, along their diagnoses and the provision of different types of radiologic services were identified using physician billing claims to OHIP. All physicians in FHGs and FHNs continue to submit fee-for-service claims to OHIP. These OHIP claims are fully paid in FHGs but only 10% of the claims are paid in FHNs representing the fee-for-service component of their blended capitation. Emergency department (ED) visits were extracted from the National Ambulatory Care Reporting System (NACRS) from the Canadian Institute for Health Information (CIHI). Prescription claims for all people over 65 years of age in Ontario were identified using the Ontario Drug Benefit Database (ODB). To identify where patients live in Ontario, a Statistics Canada's Postal Code Conversion File was used to assign postal codes of residence to 2001 census dissemination areas.

### Study Physicians and Study Patients

All physicians in Ontario who belonged to their first FHG or FHN for at least two years were identified as the study physicians. While physicians may join FHGs or FHNs at different points in time, the study timeframe was 2004 to 2007. First, we identified the FHG or FHN to which the physician joined for at least two years (i.e., stable FHG or FHN). Then we traced back in time to determine the first FHG- or FHN-free two-year period for each physician. Physicians with fewer than 100 patients rostered and FHG or FHN groups with less than three physicians were also excluded.

Patients were first selected if they were rostered to the study physician within the two years after the study physician joined their first stable FHG or FHN. Those patients that were rostered both in the post-period and had contact with the physician in the 2 year pre-period were included. Patients were excluded if they were rostered to multiple physicians or if they died within the two years after their physician joined a FHG or FHN.

### Performance Indicators

The performance measures used in this analysis were based on available administrative data, and the methods to derive these measures have been previously published [11]. The inclusion/exclusion criteria and outcome measures for each preventive care performance measure are summarized in Table 1. The eligible patient population, outcome measure and data source for the chronic disease performance measures are provided in Table 2. For the chronic disease performance measures, we identified all patients rostered to the study physicians who had a chronic disease in the Ontario Diabetes Database, the Ontario Asthma Surveillance Information System (OASIS) and by using a heart failure algorithm developed at the Institute for Clinical Evaluative Sciences (ICES). All these chronic disease databases use algorithms based on hospitalization admission data and physician visit claims data to identify both incident and prevalent cases for the entire province of Ontario. In addition all these chronic disease algorithms have been validated against physician office records [14-16].

**Table 1:** Preventive care performance measures using health administrative data

	Inclusion Criteria (Denominator)	Exclusion criteria	Outcome (Numerator)
**Cervical cancer screening**	All women aged 20 to 67 years rostered to a study physician.	Previous diagnosis of cervical cancer. Previous history of having had a hysterectomy.	Received at least one pap smear test over a two year period.

**Breast cancer screening**	All women aged 50 to 67 years rostered to a study physician.	Prior history of breast cancer.	Received at least one mammogram over a two year period.

**Colorectal cancer screening**	All men and women between 50 and 67 years of age rostered to a study physician.	Previous diagnosis of colorectal cancer or inflammatory bowel disease.	Received either a rigid or flexible sigmoidoscopy, single or double contract barium enema, colonoscopy or fecal occult blood test over a two year period.

**Table 2:** Chronic disease performance measures using health administrative data

	Eligible patient population (Denominator)	Outcome (Numerator)	Data Source
**Diabetes (DM)**			Ontario Diabetes Database

	All prevalent DM patients over 30 years of age rostered to a study physician.	Eligible DM patients who had a claim for an eye examination over a two year period.	OHIP physician encounter claims data.

	All incident DM patients rostered to a study physician over 65 years of age who started on a hypoglycemic agent.	Eligible DM patients whose first hypoglycemic agent was metformin.	ODB prescription claims data.

	All prevalent DM patients over 65 years of age rostered to a study physician.	Eligible DM patients who over one year receive a prescription for: 1) an ACEI/ARB 2) an antihypertensive agent 3) a lipid lowering agent 4) all three.	ODB prescription claims data.

**Heart Failure (HF)**			ICES Heart failure algorithm

	Incident HF patients over 40 years of age rostered to a study physician.	Eligible HF patients who received an echocardiogram within one year of diagnosis.	OHIP investigation claims data.

	Incident HF patients over 65 years of age rostered to a study physician.	Eligible HF patients who received a prescription for an ACEI or ARB.	ODB prescription claims data.

**Asthma**			Ontario Asthma Surveillance Information System

	All incident asthma patients from 20 to 40 years of age rostered to a study physician.	Eligible asthma patients who received simple spirometry or flow volume loop or bronchial provocation challenge within one of diagnosis.	OHIP investigation claims data.

	All incident asthma patients from 20 to 40 years of age rostered to a study physician.	Eligible asthma patients with emergency room visits within one year of diagnosis.	NACRS/emergency room encounter data.

Study patients with incident chronic disease were those diagnosed with that condition in the first year after the study physician joined their first stable FHN or FHG. Study patients with prevalent chronic disease were those diagnosed with a condition prior to when the study physician joined their first stable FHN or FHG.

### Stratification by age, sex and rurality

All analyses were stratified by age, sex and rurality. Rurality was defined using the Ontario Medical Association's Rurality Index of Ontario (RIO). The RIO is based on community characteristics including travel time to different levels of care; community population; presence of providers, hospitals and ambulance services; social indicators; and weather conditions [17]. RIO scores range from zero to one hundred (zero indicating the most urban and one hundred the most rural). The Ontario MOHLTC provides a rurality premium payment to FPs practicing in communities with RIO scores equal to or greater than 45. Such communities were then divided into major urban areas (RIO zero to nine), non-major urban areas (RIO ten to 44) and rural areas (RIO equal to or greater than 45).

### Analyses

For the cross-sectional study, we compared the proportion of a FHG physicians' practice who received a performance indicator to the proportion of a FHN physicians' practice who received a performance indicator. For the before-after study, statistical testing was undertaken to test the proportion of a performance indicator for a study physicians' rostered practice before the physician joined a FHG or FHN group to the proportion of a performance indicator for the study physicians' rostered practice after the physician joined a FHG or FHN. The null hypothesis is that the two proportions are the same, with p < 0.001 indicating statistical significance.

However, since the sample for the preventive care measures and some chronic disease measures is high, there is a lot of power to detect differences that may not be important at a population health or policy level. Therefore, in addition to statistical testing, we also considered a difference of more than 5% to be significant.

Data were accessed through a comprehensive research agreement between ICES and the MOHLTC. Prior to data analysis, all patient and provider identifiers were removed and replaced with unique encrypted numbers. This study was approved by the Research Ethics Board of Sunnybrook Health Science Centre in Toronto, Ontario, Canada.

## Results

The characteristics of the study population are provided in Table 3. During the study time frame (2004 to 2007), FHNs had approximately one-seventh as many FPs and one-fifth as many groups as FHGs. FHNs had a larger proportion of groups in non-major and rural areas than FHGs. There were no other statistically significant differences between FHNs and FHGs.

**Table 3:** Characteristics of study physicians practicing in primary care patient enrollment models by region and group type

		Overall	Major Urban Centres	Non-Major Urban Centres	Rural Centres
**FHG**^**‡**^	**Study Physicians (*n*)**	3466	2586	684	196
	**Groups (*n*)**	295	185	78	32
	**Male (*n*, %)**	64.6	62.8	69.3	70.9
	**Age (mean, SD)**	48.3 (9.5)	48.6 (9.4)	47.2 (9.4)	47.4 (10.1)
	**Years since graduation (mean, SD)**	25.4 (9.8)	25.7 (9.7)	24.3 (9.8)	23.9 (10.4)
	**Foreign graduation (*n*, %)**	18.8	20.2	13.7	16.8
	**Median RIO score (IQR)**	4.7 (7.6)	4.2 (3.5)	27.8 (17.1)	55.1 (11.2)
	**Total number of patients enrolled (mean, std)**	957.0 (543.8)	974.3 (541.4)	971.9 (559.2)	676.4 (435.6)
	***100-299***	335	241	57	37
	***300-649***	774	544	155	75
	***650-999***	922	701	180	41
	***1000-1499***	909	689	187	33
	***1500+***	526	411	105	10
	**Median Months in Group (IQR)**	41.5 (17.6)	40.5 (18.3)	45.0 (13.7)	42.9 (15.5)
**FHN**^**§**^	**Study Physicians (*n*)**	474	212	147	115
	**Groups (*n*)**	55	22	19	14
	**Male (*n*, %)**	66.0	59.4	70.1	73.0
	**Age (mean, SD)**	46.3 (8.7)	46.8 (8.1)	45.4 (9.2)	46.5 (9.3)
	**Years since graduation (mean, SD)**	23.6 (9.1)	24.2 (8.5)	22.6 (9.6)	23.8 (9.2)
	**Foreign graduation (n, %)**	9.7	12.3	7.5	7.8
	**Median RIO score (IQR)**	16.7 (39.2)	4.9 (2.2)	30.2 (22.4)	58.9 (18.3)
	**Total number of patients enrolled (mean, SD)**	995.4 (461.5)	1093.1 (506.9)	1000.1 (435.6)	809.2 (335.0)
	***100-299***	32	16	9	7
	***300-649***	81	27	23	31
	***650-999***	141	51	45	45
	***1000-1499***	148	73	47	28
	***1500+***	72	45	23	4
	**Median Months in Group (IQR)**	43.0 (11.0)	43.0 (12.5)	46.0 (17.0)	45.0 (7.0)

^‡ ^Family Health Group § Family Health Network

### Preventive Care

While there were statistically significant changes for cervical cancer screening after joining a FHG, there were no differences greater than 5% (Table 4). There were significant improvements after joining a FHN for the three oldest age categories across all regions. The proportion screened both in a FHG or FHN was highest in the urban areas and lowest in the rural area (p < 0.001). For both FHN and FHG patients the proportion screened decreased with age (p < 0.001). FHN patients compared to FHG patient had a higher proportion for cervical screening, especially in the rural areas (p < 0.001).

**Table 4:** Comparison of cervical cancer screening indicators before and after joining a primary care patient enrollment model by region and group type

		Overall		Major Urban Centres		Non-Major Urban Centres		Rural Centres	
		**Before**	**After**	**Before**	**After**	**Before**	**After**	**Before**	**After**

**FHG**^**‡**^	Pap test within 2 years	65.1	67.0*	66.4	68.1*^	62.0	64.1*^	53.6	56.3*^
	20-29	70.6	72.8*	70.0	72.4*	73.5	74.7*	69.2	71.5
	30-39	73.0	74.0*	73.7	74.7*^	71.8	72.2	63.7	64.8^
	40-49	68.8	70.4*^	70.1	71.7*^	65.2	67.0*^	57.8	59.8^
	50-59	61.1	61.3^	63.0	63.1^	56.3	57.0^	47.3	48.6^
	60-69	46.5	47.5*^	48.3	49.2*^	42.5	44.0*^	35.6	36.4^
**FHN**^**§**^	Pap test within 2 years	63.4	68.0*	67.6	70.7*^	61.2	66.9*^	55.1	62.2*^
	20-29	73.9	73.2	74.8	73.3	73.3	74.1	71.4	71.4
	30-39	73.2	74.3*	76.0	76.1^	71.1	72.8	67.5	70.7*^
	40-49	66.5	72.3*^	70.4	75.0*^	63.3	70.7*^	59.6	66.9*^
	50-59	57.5	62.3*^	62.4	65.5*^	54.9	60.4*^	49.2	56.9*^
	60-69	44.1	50.9*^	48.2	53.9*^	43.1	50.4*^	38.2	46.3*^

* p < 0.001 before/after comparison

^ p < 0.001 FHG/FHN comparison

^‡ ^Family Health Group

^§ ^Family Health Network

While there were statistically significant changes in mammography screening after joining a FHG, there were few changes greater than 5% (Table 5). After joining a FHN, there were statistically significant changes, but no changes over 5%, with the exception of a 5 to 10 percent increase in the proportion screened in the 60 to 69 year age group across all regions. There was a higher proportion of FHN patients compared to FHG patients who had mammography screening (p < 0.001).

**Table 5:** Comparison of breast cancer screening indicators before and after joining a primary care patient enrollment model by region and group type

		Overall		Major Urban Centres		Non-Major Urban Centres		Rural Centres	
		**Before**	**After**	**Before**	**After**	**Before**	**After**	**Before**	**After**

**FHG**^**‡**^	Mammogram within 2 years	66.6	63.3*^	66.8	63.5*^	66.1	63.2*^	64.7	59.7*^
	50-59	66.7	61.5*^	67.1	61.8*^	66.2	61.3*^	63.5	57.4*^
	60-69	66.3	66.5^	66.5	66.7^	65.9	66.5^	66.7	63.4*^
**FHN**^**§**^	Mammogram within 2 years	66.5	68.9*^	68.3	68.9^	65.8	67.5^	63.9	70.8*^
	50-59	66.8	66.6^	68.5	66.9^	65.7	64.9^	64.4	68.5*^
	60-69	66.0	72.8*^	67.8	72.7*^	66.0	71.9*^	63.1	74.0*^

* p < 0.001 before/after comparison

^p < 0.001 FHG/FHN comparison

^‡ ^Family Health Group

^§ ^Family Health Network

After joining either a FHN or FHG there was a statistically significant increase for both FHN and FHG patients in receiving any type of colorectal cancer screening (Table 6). In the rural regions, colorectal cancer screening significantly increased in FHNs compared with FHGS (p < 0.001). There were no significant differences between FHNs or FHGs in major urban or urban regions. For all regions and all age groups, the greatest increase in colorectal screening was for female patients (p < 0.001).

**Table 6:** Comparison of colorectal cancer screening indicators before and after joining a primary care patient enrollment model by region and group type

		Overall		Major Urban Centres		Non-Major Urban Centres		Rural Centres	
		**Before**	**After**	**Before**	**After**	**Before**	**After**	**Before**	**After**

**FHG**^**‡**^	**FOBT**	17.4	20.6*	18.3	21.5*^	16.3	19.5*^	8.4	10.3*^
	**Any**	24.1	30.4*^	25.1	31.6*	22.4	28.4*^	16.0	19.3*^
	**Male**	23.6	29.8*^	24.7	31.1*	21.5	27.5*^	16.2	19.4*^
	**Female**	24.6	30.9*	25.5	32.0*^	23.3	29.3*^	15.8	19.1*^
	**50-59**	22.9	28.7*^	23.9	30.0*	20.9	26.4*^	14.8	17.9*^
	**Male**	22.3	28.0*^	23.4	29.4*	19.8	25.0*^	14.8	17.9*^
	**Female**	23.5	29.4*	24.4	30.5*^	22.0	27.7*^	14.8	17.9*^
	**60-69**	26.2	33.3*^	27.2	34.5*	24.8	31.7*^	17.8	21.3*^
	**Male**	25.9	32.8*^	27.0	34.1*	24.2	31.4*^	18.3	21.6*^
	**Female**	26.5	33.6*	27.4	34.9*^	25.4	32.0*^	17.3	20.9*^
**FHN**^**§**^	**FOBT**	12.9	20.5*	14.6	23.2*^	10.0	17.9*^	12.9	18.0*^
	**Any**	19.3	29.0*^	20.8	32.0*	16.8	25.8*^	19.7	26.9*^
	**Male**	18.8	26.9*^	20.3	30.3*	16.3	23.8*^	19.1	24.1*^
	**Female**	19.8	30.9*	21.1	33.5*^	17.4	27.8*^	20.2	29.5*^
	**50-59**	18.1	27.3*^	19.4	30.3*	16.0	24.1*^	18.3	24.7*^
	**Male**	17.3	24.9*^	18.7	28.6*	15.3	21.5*^	17.1	21.4*^
	**Female**	18.9	29.4*	19.9	31.7*^	16.8	26.6*^	19.4	27.8*^
	**60-69**	21.3	31.9*^	23.4	35.3*	18.1	28.8*^	21.5	29.7*^
	**Male**	21.2	30.2*^	23.4	33.4*	17.9	27.7*^	21.5	27.6*^
	**Female**	21.4	33.5*	23.5	36.9*^	18.3	29.8*^	21.4	31.7*^

* p < 0.001 before/after comparison

^p < 0.001 FHG/FHN comparison

^‡ ^Family Health Group

§ Family Health Network

### Chronic Disease Management

Amongst people newly diagnosed with HF there was little change in the ordering of an echocardiogram within the first year of diagnosis after enrolling in a FHG (Table 7). After enrollment in a FHG, no significant change was seen with either men or women, in all age groups and in all regions. However, after enrollment in a FHN, the proportion of HF patients receiving an echocardiogram significantly increased to 50% for those 40 to 64 years of age and to 51% for those between 65 and 74 years of age. This increase in the proportion receiving an echocardiogram was higher amongst women than men in these age groups (p < 0.001). There were no differences amongst FHNs located in major urban, non-major urban or rural centres. There were no significant differences between FHNs and FHGs in their HF patients receiving an echocardiogram.

**Table 7:** Comparison of heart failure indicators before and after joining a primary care patient enrollment model, by region and group type

	Measure	Overall		Major Urban Centres		Non-Major Urban Centres		Rural Centres	
		**Before**	**After**	**Before**	**After**	**Before**	**After**	**Before**	**After**

**FHG**^**‡**^	**Echocardiogram - 1 year**								
	**40-64**	53.0	54.8	54.4	56.9	51.7	51.5^	42.2	42.5
	***Male***	55.2	56.6	56.3	59.1	54.5	53.5	45.5	40.3
	***Female***	49.6	52.0	51.5	53.6	47.4	48.6	37.9	47.2
	**65-74**	47.2	50.1	48.3	52.2	46.8	46.0	38.8	42.3
	***Male***	48.8	52.8	50.5	55.3	47.4	48.2	37.0	43.4^
	***Female***	45.3	47.1	45.6	48.9	46.1	43.6	40.5	41.1
	**75+**	37.6	38.5	38.9	40.1	36.1	36.6	30.5	28.6
	***Male***	39.9	42.3	40.6	44.9	40.2	39.8	31.8	25.0
	***Female***	35.9	35.4	37.6	36.2	33.2	34.1	29.5	31.7
	**Overall**	45.0	45.6^	46.2	47.6	43.8	42.7	36.8	35.6
	***Male***	48.1	49.2	49.2	51.8	47.6	45.8	37.9	34.3
	***Female***	41.8	41.9^	43.1	43.2	40.0	39.6	35.8	37.0
	**ACE Inhibitor - 1 year**								
	**65-74**	68.7	64.0*	67.9	64.4	70.7	62.5*^	68.5	65.8
	***Male***	73.5	69.4*	72.4	70.0	75.5	67.1*	76.5	71.1^
	***Female***	63.1	58.1*	62.6	58.2	65.0	57.2	60.7	60.3
	**75 and older**	65.3	60.6*	66.1	61.7*	63.0	59.0	66.7	55.3
	***Male***	68.9	65.4*	70.0	66.0*	66.0	63.7	68.2	65.3
	***Female***	62.8	56.7*	63.2	58.1*	60.9	55.4	65.6	46.5*
	**Overall**	66.8	61.8*	66.9	62.6*	66.3	60.2*^	67.5	59.0*
	***Male***	71.1	66.9*	71.2	67.6*	70.7	65.0*	72.2	67.5
	***Female***	62.9	57.1*	63.0	58.1*	62.4	55.9*^	63.6	51.2*
**FHN**^**§**^	**Echocardiogram - 1 year**								
	**40-64**	13.0	50.8*	49.2	59.6	42.4	40.2^	49.5	50.7
	***Male***	28.4	54.6*	51.9	65.0	42.4	44.2	48.2	52.5
	***Female***	19.0	45.5*	45.1	52.3	42.4	34.3	51.0	48.5
	**65-74**	14.8	51.4*	54.9	49.1	43.0	50.0	47.2	55.0
	***Male***	28.5	54.1*	51.3	48.3	50.0	53.3	50.8	60.0^
	***Female***	20.4	47.9*	59.1	50.0	32.6	46.0	42.6	47.7
	**75+**	31.9	34.6	36.9	35.5	25.8	32.1	29.9	36.0
	***Male***	33.7	40.5	40.9	41.3	22.6	34.7	32.1	44.0
	***Female***	30.6	30.8	33.9	31.4	27.8	30.7	28.4	30.0
	**Overall**	41.6	42.4^	45.7	43.9	36.4	38.8	40.9	44.3
	***Male***	44.3	48.3	47.8	49.6	40.1	43.7	43.6	51.0
	***Female***	38.8	37.1^	43.5	38.7	32.3	34.8	38.2	37.7
	**ACE Inhibitor - 1 year**								
	**65-74**	69.6	64.5	68.3	63.9	73.7	72.7^	67.0	56.9
	***Male***	71.9	66.5	69.7	70.0	75.0	76.7	71.2	53.8^
	***Female***	66.7	62.0	66.7	56.3	71.7	68.0	61.7	61.4
	**75 and older**	69.4	62.8	71.5	63.4	71.2	63.6	64.2	61.1
	***Male***	74.8	69.7	76.3	69.4	81.1	66.7	66.1	72.5
	***Female***	65.5	58.2	67.8	59.2	64.6	62.0	63.0	52.5
	**Overall**	69.5	63.4	70.2	63.6	72.4	66.8^	65.4	59.7
	***Male***	73.3	68.4	73.4	69.6	77.7	71.2	68.7	64.7
	***Female***	65.9	59.2	67.4	58.5	67.2	63.6^	62.5	54.9

* p < 0.001 before/after comparison

^ p < 0.001 FHG/FHN comparison

^‡ ^Family Health Group

^§ ^Family Health Network

After enrolling in a FHG or FHN, there was a statistically significant decrease of 5% to 6% in the proportion of newly diagnosed HF patients receiving a prescription for an ACEI. This slight decrease was similar amongst men and women, between different age groups and between FHGs and FHNs located in major urban, non-major urban or rural centres. There were no differences between FHNs and FHNs with respect to prescribing ACEIs.

Amongst patients newly diagnosed with DM there was a statistically significant increase with all prescribing indicators after enrolling in either a FHG or a FHN (Table 8 and Table 9). For FHGs, there was an 12% increase in people receiving a prescription for metformin, a 9% increase in receiving a prescription for a lipid lower agent, a 5% increase in receiving a prescription for an ACEI and an 8% increase in receiving all three cardiovascular medications (ACEI, lipid lower agent and antihypertensive)(p < 0.001). There was only a 3% increase in receiving an antihypertensive medication. For FHNs, there was a 15% increase in DM patients receiving a prescription for a lipid lowering agent, a 14% increase in receiving a prescription for metformin, a 10% increase in receiving a prescription for an ACEI and a 12% increased in receiving all three cardiovascular medications (ACEI, lipid lower agent and antihypertensive medication) (p < 0.001). There was a modest, though statistically significant increase with antihypertensive medication (6%) prescribing. For both FHGs and FHNs these increases were similar between men and women, at all age groups and between major urban, non-major urban and rural centres. There were no significant differences between FHNs and FHGs.

**Table 8:** Comparison of diabetes mellitus indicators before and after joining Family Health Groups by region

Measure	Overall		Major Urban Centres		Non-Major Urban Centres		Rural Centres	
	**Before**	**After**	**Before**	**After**	**Before**	**After**	**Before**	**After**

**Eye Examination - 2 years**								
**30-39**	55.6	28.7*	54.3	28.6*	59.9	28.7*	61.7	29.0*
***Male***	51.9	29.1*	50.9	29.2*	55.7	28.4*	56.5	31.0*
***Female***	58.0	28.4*	56.6	28.3*	62.5	28.9*	65.2	27.5*
**40-64**	69.8	42.6*	69.3	43.2*	71.9	41.2*	69.1	38.7*
***Male***	67.4	41.1*	67.0	41.6*	69.5	39.8*	66.5	37.6*
***Female***	72.6	44.4*	72.0	45.0*	74.9	42.8*	72.2	40.0*
**65-74**	82.7	82.6	82.1	82.0	84.6	84.5	84.8	83.9
***Male***	81.5	81.2	80.9	80.5	83.4	83.4	82.8	82.0
***Female***	84.1	84.2	83.3	83.6	86.1	85.8	87.0	86.0
**75+**	83.3	81.8	82.6	80.9	85.4	84.2	85.0	83.7
***Male***	84.0	82.5	83.4	81.6	85.7	85.0	85.4	83.8
***Female***	82.8	81.2	81.9	80.3	85.2	83.6	84.6	83.6
**Overall**	74.5	59.8*	73.7	59.3*	77.1	61.4*	76.0	60.5*
***Male***	72.8	58.4*	72.1	57.9*	75.3	60.1*	73.7	58.9*
***Female***	76.3	61.2*	75.5	60.8*	79.1	62.7*	78.5	62.3*
**Metformin - 1 year**								
**65-74**	75.3	88.3*	74.3	88.7*	78.1	87.0*	76.5	87.6*
***Male***	74.2	85.9*	72.4	86.2*	78.6	85.3*	77.2	85.5*
***Female***	76.3	90.4*	76.0	91.0*	77.6	88.8	75.6	89.4*
**75 and older**	73.9	84.1*	73.8	83.4*	74.4	84.4*	72.3	90.3*
***Male***	75.5	84.3*	75.0	83.0	78.9	86.8*	64.3	89.7*
***Female***	72.8	83.9*	73.0	83.7*	71.5	82.6*	75.8	90.7*
**Overall**	74.8	86.7*	74.1	86.7*	76.8	86.0*	75.2	88.6*
***Male***	74.6	85.4*	73.2	85.1*	78.7	85.8*	74.6	86.9*
***Female***	75.0	87.8*	75.0	88.1*	75.1	86.2*	75.7	89.9*
**ACE inhibitor - 1 year**								
**65-74**	67.5	72.5*	67.6	72.5*	66.9	73.0*	67.6	72.0*
***Male***	68.0	73.5*	68.2	73.4*	67.6	73.9*	67.4	72.8*
***Female***	66.9	71.5*	67.0	71.4	66.2	71.8	67.8	71.0
**75 and older**	66.1	71.5*	66.6	72.0*	64.9	70.6	64.2	68.4
***Male***	65.8	71.3*	66.4	71.9	64.6	70.2	62.9	67.1
***Female***	66.4	71.8*	66.8	72.2	65.2	70.9	65.3	69.6
**Overall**	67.0	72.1*	67.3	72.3*	66.2	71.9*	66.3	70.4*
***Male***	67.3	72.6*	67.6	72.8*	66.6	72.5*	65.8	70.5*
***Female***	66.7	71.6*	66.9	71.7*	65.7	71.4*	66.7	70.4*
**Antihypertensive agent - 1 year**								
**65-74**	64.9	67.7	64.4	67.1*	65.6	69.0*	68.3	70.7
***Male***	63.0	66.2	62.7	65.8*	63.4	66.8*	66.2	69.0
***Female***	66.9	69.4	66.4	68.6	68.1	71.7	70.6	72.8
**75 and older**	73.8	76.6*	73.5	76.2	74.2	77.1	76.8	80.0*
***Male***	70.6	74.0	70.4	73.7	70.6	74.1	73.1	77.3
***Female***	76.3	78.7	75.9	78.3	76.9	79.5	79.8	82.3
**Overall**	68.2	71.4*	67.7	70.9*	68.9	72.5*	71.6	74.8*
***Male***	65.5	69.2*	65.2	68.8*	65.8	69.6*	68.6	72.3*
***Female***	70.8	73.7*	70.2	72.9*	71.9	75.5*	74.6	77.3
**Lipid-lowering agent - 1 year**								
**65-74**	59.3	67.9*	59.9	68.5*	57.5	66.4*	57.2	65.5*
***Male***	60.2	69.0*	60.7	69.4*	58.9	68.0*	58.5	67.3*
***Female***	58.2	66.7*	59.0	67.4*	56.0	64.4	55.8	63.3
**75 and older**	50.1	59.4*	52.2	60.8	44.4	55.8	45.1	53.7
***Male***	51.0	61.1*	52.8	62.3*	46.2	58.1	46.3	56.4
***Female***	49.5	58.0*	51.8	59.6	43.0	54.0	44.1	51.4
**Overall**	55.9	64.3*	57.1	65.3	52.5	61.8*	52.5	60.3*
***Male***	57.2	66.0*	58.1	66.7*	54.7	64.2*	54.2	62.9*
***Female***	54.6	62.7*	56.1	63.9	50.4	59.4*	50.8	57.6*
**All three - 1 year**								
**65-74**	35.4	43.0*	35.7	43.2*	34.0	42.4*	36.8	43.0*
***Male***	36.0	43.9*	36.3	44.2	34.7	43.0*	37.0	43.8
***Female***	34.8	42.0*	35.1	42.1*	33.3	41.6	36.5	42.0*
**75 and older**	32.4	40.4*	33.7	41.5	28.6	38.1*	28.5	35.5
***Male***	32.4	41.0*	33.6	42.1*	29.1	38.6	28.6	36.1*
***Female***	32.4	39.9*	33.9	41.0	28.2	37.6	28.5	35.0
**Overall**	34.3	41.9*	35.0	42.5*	32.0	40.5*	33.6	39.7*
***Male***	34.8	42.8*	35.4	43.4*	32.8	41.3*	34.0	40.7*
***Female***	33.8	41.1*	34.6	41.6*	31.1	40.0*	33.1	38.7*

* p < 0.001 before/after comparison.

**Table 9:** Comparison of diabetes mellitus indicators before and after joining Family Health Networks by region

Measure	Overall		Major Urban Centres		Non-Major Urban Centres		Rural Centres	
	**Before**	**After**	**Before**	**After**	**Before**	**After**	**Before**	**After**

**Eye Examination - 2 years**								
**30-39**	64.4	34.1*	66.8	35.3*	62.0	35.6*	61.4	28.5*
***Male***	59.4	34.6*	63.2	34.5*	58.6	36.7*	51.7	31.6*
***Female***	68.0	33.8*	69.3	35.9*	64.8	34.9*	68.8	26.5*
**40-64**	73.9	46.1*	74.4	45.8*	73.4	46.9*	73.6	45.6*
***Male***	71.5	45.2*	72.1	45.1*	71.5	46.6*	70.3	43.5*
***Female***	76.9	47.2*	77.2	46.6*	75.9	47.3*	77.5	48.0*
**65-74**	83.9	85.5*	84.4	85.6	82.6	85.9	84.5	84.8
***Male***	82.4	84.1	82.6	84.1	81.7	84.5	82.9	83.6
***Female***	85.6	87.1	86.5	87.3	83.7	87.6	86.3	86.3
**75+**	86.0	84.0	85.7	84.7	85.9	83.6	86.5	83.2
***Male***	85.2	85.6	84.1	85.8	86.0	86.9	86.3	83.9
***Female***	86.5	82.6	86.8	83.8	85.9	80.9	86.7	82.6
**Overall**	78.2	64.6*	78.4	63.4*	77.5	64.7*	78.5	65.7*
***Male***	76.0	63.6*	76.1	62.4*	74.0	64.3*	75.9	65.0*
***Female***	80.5	65.1*	80.8	64.4*	79.4	65.2*	81.3	66.5*
**Metformin - 1 year**								
**65-74**	68.5	86.7*	64.8	86.6*	77.8	85.1*	66.2	88.2*
***Male***	77.0	84.3*	77.5	83.3	81.8	87.5	73.7	82.9
***Female***	61.2	89.5*	54.9	90.7*	75.0	82.9	57.6	96.3
**75 and older**	69.2	83.2*	66.1	82.8*	64.5	83.6*	80.0	83.3
***Male***	73.1	80.6*	69.2	71.4	75.0	88.0	78.6	81.3
***Female***	66.2	84.9*	63.3	88.4*	57.9	80.0	81.3	85.0
**Overall**	68.8	85.3*	65.3	85.1*	72.9	84.4*	70.3	86.5*
***Male***	75.7	83.1*	74.2	80.0	79.4	87.7	75.0	82.5
***Female***	63.0	87.4*	58.0	89.5*	68.6	81.5	65.3	91.5*
**ACE inhibitor - 1 year**								
**65-74**	61.2	71.1*	61.1	70.9*	63.4	72.6*	59.0	69.7*
***Male***	62.0	71.7*	61.6	71.7*	64.4	73.6*	59.9	69.9*
***Female***	60.3	70.2*	60.5	70.0*	62.2	71.4	57.9	69.4*
**75 and older**	58.9	68.9*	58.5	69.5*	60.3	70.2*	58.0	66.5*
***Male***	57.4	69.1*	55.8	69.7*	61.1	71.8	55.8	65.2*
***Female***	60.0	68.8*	60.4	69.3*	59.8	68.9*	59.7	67.6
**Overall**	60.4	70.1*	60.1	70.3*	62.2	71.5*	58.6	68.4*
***Male***	60.5	70.7*	59.8	70.9*	63.4	72.9*	58.7	68.2*
***Female***	60.2	69.5*	60.5	69.7*	61.1	70.2*	58.6	68.5*
**Antihypertensive agent - 1 year**								
**65-74**	64.9	68.0*	63.8	68.0*	68.1	69.2	63.0	66.7
***Male***	61.2	65.7*	60.9	66.4	64.5	67.1	58.2	63.3
***Female***	68.5	70.7*	66.7	69.8	71.5	71.7	68.0	71.1
**75 and older**	71.0	75.0*	69.0	74.5*	75.7	76.1	68.6	74.5
***Male***	67.4	72.2*	65.9	71.8	72.6	71.6	64.2	73.5
***Female***	73.6	77.3*	71.3	76.6*	77.9	79.7	72.1	75.5
**Overall**	64.9	71.1*	63.8	71.0*	68.1	72.2*	63.0	69.9*
***Male***	61.2	68.2*	60.9	68.6*	64.5	68.9*	58.2	67.0*
***Female***	68.5	73.9*	66.7	73.2*	71.5	75.6*	68.0	73.1
**Lipid-lowering agent - 1 year**								
**65-74**	47.3	63.5*	50.5	67.2*	47.0	60.8*	42.9	60.6*
***Male***	47.8	64.8*	51.9	69.6*	46.3	61.9*	43.2	61.0*
***Female***	46.7	61.8*	48.8	64.4*	47.8	59.4*	42.5	60.2*
**75 and older**	38.5	53.2*	40.5	56.8	37.0	49.8*	36.7	50.6
***Male***	40.9	57.6*	41.3	59.5*	41.9	56.2*	39.2	55.9
***Female***	36.7	49.7*	39.9	54.8	33.7	44.7	34.7	46.0
**Overall**	44.1	58.9*	46.7	62.5*	43.2	55.9*	40.8	56.5*
***Male***	45.7	62.0*	48.5	65.5*	45.0	59.7*	42.0	59.1*
***Female***	42.5	55.9*	45.0	59.6*	41.5	52.1*	39.5	53.6*
**All three - 1 year**								
**65-74**	25.7	39.5*	27.2	41.7*	26.4	39.1*	22.8	36.5*
***Male***	25.7	40.1*	27.9	42.8*	25.8	40.3*	22.3	36.2*
***Female***	25.7	38.7*	26.3	40.4*	27.1	37.8*	23.3	36.8*
**75 and older**	22.5	35.4*	23.9	37.8*	21.7	33.9*	21.0	33.0
***Male***	23.3	37.4*	22.1	38.0*	25.5	37.3*	22.8	36.5*
***Female***	21.9	33.8*	25.1	37.6*	19.1	31.1	19.5	29.9
**Overall**	24.5	37.7*	25.9	39.9*	24.6	36.8*	22.2	35.0
***Male***	24.9	39.1*	26.0	40.9*	25.7	39.1*	22.4	36.3*
***Female***	24.1	36.3*	25.8	39.0*	23.5	34.5*	21.9	33.6

* p < 0.001 before/after comparison

After enrolling in a FHG there was an overall 15% decrease and after enrolling in a FHN a 14% decreased in the proportion of DM patients having an annual eye examination (p < 0.001). This decrease was highest amongst people with DM less than 65 years of age (27% and 29%) compared with people over 65 years of age (about 2%). The decrease was similar between men and women and between major urban, non-major urban and rural centres.

After joining either a FHG or FHN, for people with newly diagnosed asthma (Table 10) there were no statistically significant changes in spirometry testing and emergency department (ED) visits within one year of diagnosis. There was no statistically significant change after joining a FHG of FHN for both men and women, by all age groups and for FHG practices in major urban, non-major urban and rural centres. There were no significant differences between FHGs and FHNs for the asthma performance measures.

**Table 10:** Comparison of asthma indicators before and after joining a primary care patient enrollment model, by region and group type

	Measure	Overall		Major Urban Centres		Non-Major Urban Centres		Rural Centres	
		**Before**	**After**	**Before**	**After**	**Before**	**After**	**Before**	**After**
**FHG‡**	**Spirometry test - one year**								
	**10 to 19**	32.1	34.2	31.9	34.6	31.8	33.5	37.2	30.4^
	***Male***	30.9	34.0	30.0	33.8	32.3	35.6	39.0	28.6
	***Female***	32.8	34.4	33.1	35.3	31.5	32.2	35.8	31.8^
	**20 to 29**	31.7	33.7	33.1	33.2	28.1	36.0*	19.2	34.1
	***Male***	32.8	33.0	33.4	32.9	30.9	33.8	26.7	31.3
	***Female***	31.1	34.1	32.9	33.3	27.1	37.0*	16.2	36.0
	**30 to 39**	33.0	33.4	31.6	32.3	39.7	40.1	26.3	20.4
	***Male***	34.4	34.7	32.2	34.2	45.1	37.9	22.2	33.3
	***Female***	32.2	32.7	31.2	31.3	36.9	41.1	27.6	17.5
	**Overall**	32.3	33.8	32.1	33.4	33.5	36.2	29.3	28.4^
	***Male***	32.7	34.0	31.7	33.7	36.3	35.9	32.4	30.0
	***Female***	32.1	33.6	32.3	33.2	32.1	36.4	27.7	27.5^
	**Emergency Department Visit**								
	**10 to 19**	7.9	8.4	5.9	6.7	12.7	11.8	14.9	19.0
	***Male***	6.6	7.8	4.5	6.1	11.8	11.0	12.2	20.0
	***Female***	8.7	8.8	6.8	7.2	13.3	12.3	17.0	18.2
	**20 to 29**	10.4	8.2^	9.5	6.7*	12.8	12.3	17.3	24.4
	***Male***	10.0	6.8	8.2	4.8*	16.0	11.3	26.7	31.3
	***Female***	10.5	8.9	10.1	7.6^	11.7	12.7	13.5	20.0
	**30 to 39**	8.0	7.2^	6.4	6.0	13.1	10.0	18.4	28.6
	***Male***	6.9	6.4	5.5	5.9	11.6	7.8	16.7	22.2
	***Female***	8.6	7.6	6.8	6.0	13.8	11.0	19.0	30.0
	**Overall**	8.6	8.0^	7.0	6.5^	12.8	11.4	16.7	23.1
	***Male***	7.5	7.2	5.8	5.9	12.5	10.1	16.2	23.3
	***Female***	9.2	8.4^	7.7	6.9^	13.0	12.1	16.9	22.9
**FHN§**	**Spirometry test - one year**								
	**10 to 19**	35.8	39.4	30.8	36.8	40.0	36.1	42.9	52.4^
	***Male***	31.0	37.1	24.2	39.1	38.7	32.1	38.9	40.0
	***Female***	39.6	40.8	36.5	35.0	41.0	38.2	44.7	59.3^
	**20 to 29**	31.7	37.9	27.2	37.5	32.8	34.5	44.4	46.7
	***Male***	40.3	28.6	38.5	18.8	31.8	37.5	63.6	50.0
	***Female***	27.2	40.9	21.3	42.9*	33.3	33.3	36.0	45.5
	**30 to 39**	37.1	39.9	36.7	42.1	33.7	36.5	44.7	38.1
	***Male***	43.8	37.7	41.1	40.7	48.3	31.6	45.0	42.9
	***Female***	33.5	40.9	34.5	42.6	26.3	39.4	44.4	35.7
	**Overall**	35.3	39.2	32.0	38.8	36.7	36.0	43.9	47.4^
	***Male***	36.8	35.9	32.3	36.0	39.8	32.7	46.9	42.3
	***Female***	34.4	40.9	31.8	40.2	34.7	37.6	42.2	50.0^
	**Emergency Department Visit**								
	**10 to 19**	11.1	10.4	7.2	6.6	13.1	15.7	19.6	9.5
	***Male***	13.5	6.7	9.9	6.5	17.7	7.1	16.7	6.7
	***Female***	9.3	12.7	4.8	6.7	9.6	20.0	21.1	11.1
	**20 to 29**	14.9	19.8^	10.5	19.4	13.8	13.8	30.6	33.3
	***Male***	2.8	10.7		6.3	4.5	12.5	9.1	25.0
	***Female***	21.3	22.7	16.0	23.2^	19.4	14.3	40.0	36.4
	**30 to 39**	10.9	13.7^	5.9	9.5	14.0	15.4	23.4	28.6
	***Male***	14.3	17.0	8.9	7.4	20.7	21.1	20.0	42.9
	***Female***	9.1	12.2	4.4	10.3	10.5	12.1	25.9	21.4
	**Overall**	11.9	14.6^	7.5	11.7^	13.5	15.9	23.7	21.8
	***Male***	11.5	11.2	7.5	6.7	15.9	14.5	16.3	19.2
	***Female***	12.2	16.2^	7.5	14.1*^	11.9	16.5	27.8	23.1

* p < 0.001 before/after comparison

^p < 0.001 FHG/FHN comparison

^**‡ **^**Family Health Group**

^**§ **^**Family Health Network**

## Discussion

Several factors may influence chronic disease management in family medicine. For example, practices located in rural regions are challenged by less availability and access to technology or specialty care to help diagnose or monitor some conditions. For chronic diseases, this may affect some aspects of patient care such as echocardiogram and spirometry testing and ophthalmologic assessment. Physician knowledge, experience or comfort in managing chronic disease may influence medical therapy that their patients receive, such as medications. Practice structures can facilitate chronic disease management through processes such as interdisciplinary care. Physician remuneration models and pay for performance incentives may improve benchmark levels of chronic disease performance measures.

The results for the HF and DM prescribing indicators are similar to those measured in other studies conducted in Canada [11,18,19]. Benchmark prescribing levels for HF patients are usually based on patients discharged from hospital and do not include HF patients diagnosed and managed outside of the hospital setting [20]. Quality of care for DM management has focused on clinical targets and less so on providing specific prescribing benchmarks [21]. Nevertheless, the prescribing levels for HF and DM patients in this study approach some evidence based targets. While the HF and DM prescribing indicators did not significantly differ from patients belonging to a FHG versus a FHN, some prescribing indicators did differ slightly (of around 5%) by region in Ontario. However, further work which would control for potential confounders such as socioeconomic status and comorbidity still needs to be done to confirm these comparisons.

Since 2002, several clinical evidence-based guidelines have been disseminated to primary care practitioners for DM management [21,22]. In this analysis, after patients enrolled in either a FHG or FHN model, improvements were seen in the prescribing of metformin, angiotensin converting enzyme inhibitor (ACEI) and antilipid medications. This may be a result of incentive payments for DM care. It may also reflect the success in knowledge translation of evidence based care for DM. Interestingly, while the prescribing of antihypertensive medications had not reached benchmark levels, we did not see a significant change after patients enrolled in either FHGs or FHNs.

In this analysis, there were no significant changes in ACEI prescribing for HF patients after they enrolled in either a FHG or FHN model. The HF management incentives for FHGs and FHNs were introduced in 2008, after the study time frame. Although evidence-based guidelines have been developed by the Canadian Cardiology Association for the management of HF patients, their dissemination into primary care practice has been limited [20]. Rather than concluding a lack of affect of primary care delivery models affecting HF management, our study may point to a lack of knowledge translation of the current HF recommendations into primary care. Follow up work, after the HF management incentives were implemented, may better demonstrate any potential impact they may have in the care of HF patients.

More striking gender and age differences were found. We found lower echocardiogram use and ACEI prescribing for women newly diagnosed with HF and lower ophthalmology use for DM patients less than 64 years of age. For several reasons, women receive fewer cardiovascular investigations than men [23,24]. As of November 1 2004, funding for routine eye examinations by either an optometrist or physician for patients between 20 and 64 years of age was no longer covered under the publically funded health insurance plan. However, patients of all ages with stipulated medical conditions, such as DM are still eligible for an annual eye examination. It may be that younger DM patients and their FPs are unaware of this coverage and this may be one reason for poorer referrals for younger DM patients.

There were no improvements in our performance measures for asthma care and no asthma management incentives exist for either model. While asthma guidelines exist for FPs, the emphasis on evidence-based clinical care often focuses on the medications prescribed for people with asthma and less so on health administrative data indicators such as spirometry testing and emergency room use as used in this study [25]. Further analysis should include an examination of medication use by people with asthma.

While the proportion or women getting mammography or pap smear testing was higher amongst those belonging to a FHN versus a FHG, the differences were not large. The capitation remuneration payment for FPs participating in FHN models may account for this slight improvement. In Ontario, benchmark mammography and pap smear testing levels are generally set at 75% for screen eligible women [26]. In this study, both mammography and pap smear testing met or approached these levels, regardless of the physician remuneration structure or practice location. However, secular trends in Ontario for mammography and pap smear testing prior to the introduction of these new primary care models were already approaching benchmark levels [11]. Although the proportion of the study patients receiving colorectal screening is still low, it did improve significantly after patients joined either a FHG and FHN, and in comparison to similar provincial results released in 2006 [11]. No differences were seen in colorectal screening between FHGs and FHNs. In March 2008, after the time frame of this study, a new Cancer Care Ontario colorectal screening program was launched, which included more financial incentives to improve colorectal screening benchmarks [27]. Further comparison with primary care models which do not have incentive payments may expand the understanding of the impact of these initiatives on colorectal cancer screening rates. In England, an examination of 18 general practices found that financial incentives introduced to improve quality of care for "incentivized" conditions and non-"incentivized" conditions did not demonstrate any quality improvement [28]. Similarly another study, based in the UK, found that the quality of care for asthma, diabetes and coronary artery disease was improving before the introduction of 2004 pay for performance incentives. However, it did conclude there was a modest acceleration in quality improvement for diabetes and asthma after 2004 [29]. Another British study, which examined preventive prescribing indicators and the health gains related to potential payments (including incentive payments), found no relationship between pay and health gain across the prescribing interventions examined [30].

A retrospective review of a US Medicare community health centre population which used administrative data to measure performance did not find evidence of clinically significant change with financial incentives for preventive care performance [31]. Another US study of 35 Kaiser Permanente facilities found the removal of financial incentives was associated with decreased DM retinopathy screening and cervical cancer screening [32].

A systematic review of studies examining pay for performance confirms that the results of pay for performance range from extremely positive to disappointing [33]. Among the recommendations made to ensure success with the selection of pay for performance incentives are the selection and definition of pay for performance targets on the basis of baseline room for improvement. This may be the situation for breast cancer and cervical cancer screening activity in Ontario. Prior to the introduction of FHGs and FHNs, these screening rates were approaching 75% [11]. Colorectal cancer screening rates were extremely low prior to the introduction of FHNs and FHG, and therefore incentive payments, along with other provincial strategies, may have contributed to the improvements seen in screening rates.

There are several limitations to our study. First, this study was limited to enrolled patients, and as the numbers continue to rise as more patients enroll in FHGs, FHNs and other models further research would be warranted. In some cases, the period of time over which the indicator was measured was insufficient. For example, a two and half year window for determining mammography screening may be more appropriate than two years. Using administrative data alone poses challenges in assessing quality of care. For example, getting a prescription for a medication is not the same as actually taking it. And finally tracking FP care is challenging in Canada, as FPs may participate in more than one type or primary care model.

## Conclusions

Some improvements in preventive screening and DM management were seen amongst people after they enrolled in a FHG or FHN. FHNs, a capitation-based model, demonstrated some improvements in care, especially in rural regions. To some degree these improvements may be attributed to incentive payments offered within FHGs and FHNs. However, these primary care delivery models need to be compared with other primary care delivery models such as Family Health Organizations (FHOs), Family Health Teams (FHTs), Community Health Centres (CHCs) and fee for service practices in order to fully describe more specifically what aspects of model delivery and incentives affect care.

## Competing interests

The authors declare that they have no competing interests.

## Authors' contributions

All authors were involved in the conception and design of the study. AK was primarily responsible for data abstraction and analysis, with additional contributions from JG. JG and LJ performed the statistical analysis. LJ, JG and JB prepared the first draft of the article, and RG, LJ and JG were responsible for subsequent revisions. All authors critically reviewed the article and approved the final version.

## Pre-publication history

The pre-publication history for this paper can be accessed here:

http://www.biomedcentral.com/1471-2296/12/44/prepub

## References

[B1] Starfield B Griffin J Is Strong Primary Care Good for Health Outcomes? The Future of Primary Care: Papers for a Symposium held on 13th September 1995 1996 London: Office of Health Economics 18 29

[B2] The World Health Report 2008: Primary care now more than ever 2008 World Health Organization http://www.who.int/whr/2008/whr08_en.pdf accessed May 18, 2011

[B3] Browne G Roberts J Gafni A Byrne C Weir R Majumdar B Watt S Economic evaluations of community-based care: lessons from twelve studies in Ontario J Eval Clin Pract 1999 5 4 367 85 10.1046/j.1365-2753.1999.00191.x 10579701

[B4] National Health Service (Primary Care) Act 1997, Chapter 46 1997 National Health Service, London http://www.legislation.gov.uk/ukpga/1997/46/part/II accessed April 9, 2011

[B5] Primary Health Care Reform in Australia: Report to Support Australia's First National Primary Health Care Strategy 2009 Department of Health and Ageing, Australian Government, Commonwealth of Australia http://www.yourhealth.gov.au/internet/yourhealth/publishing.nsf/Content/nphc-draftreportsupp-toc/$FILE/NPHC-supp.pdf accessed April 9, 2011

[B6] Health Care and Education Reconciliation Act of 2010 http://www.gpo.gov/fdsys/pkg/PLAW-111publ152/pdf/PLAW-111publ152.pdf Public Law 111-152. accessed April 9, 1011

[B7] Romanow RJ Building on Values: the future of health care in Canada - Final Report. Commission on the future of health care in Canada 2002 National Library of Canada http://dsp-psd.pwgsc.gc.ca/Collection/CP32-85-2002E.pdf accessed April 9, 2011

[B8] Gosden T Forland F Kristiansen IS Sutton M Leese B Giuffrida A Sergison M Pedersen L Impact of payment method on behaviour of primary care physicians: a systematic review J Health Serv Res Policy 2001 6 1 44 55 10.1258/1355819011927198 11219360

[B9] Canadian Institute for Health Information Pan-Canadian Primary Health Care Indicators. Pan-Canadian Primary Health Care Indicators Report 2006; Report 1 2006 1 http://www.cihi.ca/CIHI-ext-portal/pdf/internet/PDF_PHC_INDI_REPORT1VOL1FIN_EN accessed April 9, 2011

[B10] Levitt C Hilts L Quality in family practice book of Tools 2010 Hamilton: McMaster Innovation Press

[B11] Jaakkimainen L Klein-Geltink JE Guttmann a Barnsley J Zagorski BM Kopp A Saskin R Leong A Wang L Jaakkimainen L, Upshur REG, Maaten S, Schultz SE Chapter 12 - Indicators of Primary Care Based on Administrative Data Primary Care in Ontario: ICES Atlas. Institute for Clinical Evaluative Sciences 2006 Toronto 177 250 http://www.ices.on.ca/file/CHP12_erratum_May29_2009.pdf accessed April 9, 2011

[B12] Katz A DeCoster C Bogdanovic B Soodeen RA Chateau D Using Administrative Data to Develop Indicators of Quality in Family Practice 2004 Manitoba Centre for Health Policy, Manitoba Health http://mchp-appserv.cpe.umanitoba.ca/reference/quality_wo.pdf accessed April 9, 2011

[B13] Stand up to Diabetes About the Ontario Diabetes Strategy. Queen's printer for Ontario 2009 http://www.health.gov.on.ca/en/ms/diabetes/en/about_diabetes_strategy.html accessed May 18, 2011

[B14] Hux JE Flintoft V Ivis F Bica A Diabetes in Ontario - Determination of prevalence and incidence using a validated administrative data algorithm Diabetes Care 2002 25 3 512 6 10.2337/diacare.25.3.512 11874939

[B15] Ezekowitz JA Kaul P Bakal JA Quan H McAlister FA Trends in heat failure care: has the incident diagnosis of heart failure shifted from the hospital to the emergency department and outpatient clinics? Eur J Heart Fail 2011 13 2 142 7 10.1093/eurjhf/hfq185 20959343

[B16] Gershon AS Wang C Guan J Vasilevska-Ristovska J Cicutto L To T Identifying patients with physician-diagnosed asthma in health administrative databases Can Resp J 2009 16 6 183 188 10.1155/2009/963098 PMC2807792 20011725

[B17] Kralj B Measuring "rurality" for purposes of health-care planning: an empirical measure for Ontario Ont Med Rev 2000 33 52

[B18] Jackevicius CA Tu K Filate WA Brien SE Tu JV Trends in cardiovascular drug utilization and drug expenditures in Canada between 1996 and 2001 Can J Cardiol 2003 19 12 1359 66 14631469

[B19] Shah B Mamdani M Kopp A Hux JE, Booth GL, Slaughter PM, Laupacis A Chapter 3. Drug Use in Older People with Diabetes Diabetes in Ontario: An ICES Practice Atlas. Institute for Clinical Evaluative Sciences 2003 2.21 2.51 http://www.ices.on.ca/file/DM_Chapter3.pdf accessed April 9, 2011

[B20] Canadian Cardiovascular Outcomes Research Team Quality of Cardiac Care in Ontario. EFFECT (Enhances Feedback for Effective Cardiac Treatment) Phase 1. Report 1. Institute for Clinical Evaluative S22. Lee DS, Tran C, Flintoft V, Grant FC, Liu PP, Tu JV and the Canadian Cardiovascular Outcome Research Team. CCORT/CCS quality indicators for congestive heart failure care Can J Cardiol 2003 19 4 357 364 12704479

[B21] Majumdar SR Johnson JA Bowker SL Booth GL Dolovich L Ghali W Harris SB Hux JE Holbrook A Lee HN Toth EL Yale J-F A Canadian Consensus for the Standardized Evaluation of Quality Improvement Interventions in Type 2 Diabetes Can J Diabetes 2005 29 3 220 229

[B22] Canadian Diabetes Association Clinical Practice Guidelines Expert Committee Canadian Diabetes Association 2008. Clinical practice guidelines for the prevention and management of diabetes in Canada Can J Diabetes 2008 32 suppl 1 vi ix 10.1016/j.jcjd.2013.01.009 24070926

[B23] Bierman AS Jaakkimainen RL Abramson BL Kapral MK Azad N Hall R Lindsay P Honein G Degani N Bierman AS Cardiovascular Disease Project for an Ontario Women's Health Evidence-Based Reprot 2009 1 Toronto http://www.powerstudy.ca/the-power-report/the-power-report-volume-1/cardiovascular-disease accessed April 9, 201121350259

[B24] Burstein JM Yan R Weller I Abramson BL Management of congestive heart failure: a gender gap may still exist Observation from a contemporary cohort. BMC Cardiovascular Disorders 2003 3 1 1 7 http://www.biomedcentral.com/1471-2261/3/1/accessed May 18, 2011 10.1186/1471-2261-3-1 PMC149453 12590653

[B25] Becker A Lemiere C Berube D Boulet LP Ducharme FM FitzGerald M Kovesi T on behalf of the Asthma Guidelines Working Group of the Canadian Network for Asthma Care and the Canadian Thoracic Society Summary of the recommendations from the Canadian Asthma Consensus Guidelines, 2003 CMAJ 2005 1733 6 S3 S7 PMC1329945 16157733

[B26] Cancer Care Ontario Information for Primary Care Providers on the Ontario Breast Screening Program (OBSP) 2008 http://www.cancercare.on.ca/pcs/screening/breastscreening/resources/accessed December 31, 2010

[B27] Colon Cancer Check Key Features and Resources for Primary Care Physicians http://health.gov.on.ca/en/ms/coloncancercheck/accessed December 31, 2010

[B28] Steel N Maisey S Clark A Fleetcroft R Howe A Quality of clinical primary care and targeted incentive payments: an observational study Br J Gen Pract 2007 57 539 449 454 17550669 PMC2078183

[B29] Campbell A Reeves D Kontopantelis E Middelton E Sibbald B Roland M Quality of Primary Care in England with the Introduction of Pay for Performance N Engl J Med 2007 357 2 181 90 10.1056/NEJMsr065990 17625132

[B30] Fleetcroft R Cookson R Do the incentive payments in the new NHS contract for primary care reflect likely population health gains? J Health Serv Res Policy 2006 11 1 27 31 10.1258/135581906775094316 16378529

[B31] Gavagan TF Du H Saver BG Adams GJ Graham DM McCray R Goodrick GK Effect of financial incentives on improvement in medical quality indicators for primary care J Am Board Fam Med 2010 23 5 622 31 10.3122/jabfm.2010.05.070187 20823357

[B32] Lester H Schmittdiel J Selby J Fireman B Campbell S Lee J Whippy A Madvig P The impact of removing financial incentives from clinical quality indicators: longitudinal analysis of four Kaiser Permanente indicators BMJ 2010 340 C1898 10.1136/bmj.c1898 20460330 PMC2868163

[B33] Van Herck P De Smedt D Annemans L Remmen R Rosenthal MB Sermeus W Systematic review: Effects, design choices, and context of pay-for-performance in health care BMC Health Serv Res 2010 10 247 review 10.1186/1472-6963-10-247 20731816 PMC2936378

